# Phytochemical Contents and Antioxidant Capacities of Two *Aloe greatheadii* var. *davyana* Extracts

**DOI:** 10.3390/molecules13092169

**Published:** 2008-09-12

**Authors:** Lisa Botes, Francois H. van der Westhuizen, Du Toit Loots

**Affiliations:** 1 School for Physiology, Nutrition and Consumer’s Science, Private Bag X6001, Box 594, Potchefstroom, 2531, South Africa; E-mail: 12167398@nwu.ac.za (Lisa Botes); 2 School for Physical and Chemical Sciences, North-West University, Private Bag X6001, Box 269, Potchefstroom, 2531, South Africa; E-mails: francois.vanderwesthuizen@nwu.ac.za (Francois H. van der Westhuizen)

**Keywords:** Phytochemical content, antioxidant capacity, polyphenols, phytosterols

## Abstract

*Aloe greatheadii* var. *davyana* (Asphodelaceae) is used among rural South African communities to treat arthritis, skin cancer, burns, eczema, psoriasis, digestive problems, high blood pressure and diabetes, despite very little supporting scientific evidence. Due to increased interest by both the scientific community and industry regarding the medicinal uses of this plant species, we identified, quantified and compared the phytochemical contents and antioxidant capacities of two extracts of *A. greatheadii;* a leaf gel extract (LGE) and a 95 % aqueous ethanol leaf gel extract (ELGE), using various modified extraction procedures, GC-MS and spectrophotometry. Apart from extensively characterizing this medicinal plant with regards to its organic acid, polyphenols/phenolic acid, alcohol, aldehyde, ketone, alkane, pyrimidine, indole, alkaloid, phytosterol, fatty acid and dicarboxylic acid contents and antioxidant capacities, we describe a modified extraction procedure for the purpose of general phytochemical characterization, and compare this to a 95 % aqueous ethanol extraction technique. From the results it is clear that *A. greatheadii* contains a variety of compounds with confirmed antioxidant capacity and other putative health benefits (such as blood glucose, cholesterol and cortisol lowering properties) relating to the prevention or treatment of diabetes, cardiovascular disease, cancer and hypertension. The results also indicate that separate ethyl acetate/diethyl ether and hexane extractions of the LGE, better serve for general phytochemical characterization purposes, and 95 % aqueous ethanol extraction for concentrating selective groups of health related compounds, hence justifying its use for biological *in vivo* efficacy studies.

## Introduction

Populations in developing countries worldwide rely heavily on the use of traditional medicine as their primary source of healthcare [1]. In South Africa alone over 27 million people use indigenous plant based medicines and up to 60 % of the population consult with one of 200 000 traditional healers [2]. Additionally, there is an increased global commercial interest in the use of these plant species for their proposed health benefits despite little or no scientific evidence justifying the anecdotal claims accompanying many of these products.

*Aloes* have been used therapeutically since ancient times [3,4] and interest in the inner, colourless leaf gel has increased over the last two decades [5]. Partial characterization and biological action of various extracts of the leaf gel have been described, especially pertaining to diabetes [6]. The majority of the scientifically based research on this topic has to date, however, been done exclusively on two *Aloe* species namely; *Aloe vera* (or *Aloe barbadensis*) and *Aloe arborescens*. *Aloe* gel (from a variety of *Aloe* species) is sold commercially worldwide as an ingredient to a wide range of health care, cosmetic and therapeutic products [6]. This commercial activity, and widely distributed use of *Aloe* as used in traditional medicine, has led to an upsurge of both clinical and chemical research focusing on the active ingredients in these plants, as well as their biological activities.

The species of *Aloe* selected for commercial exploitation or selected by the traditional healer, would be based on its local availability and distribution. In South Africa the most widely distributed *Aloe* species are *Aloe greatheadii* var. *davyana* (Asphodelaceae) and *Aloe ferox* Mill. (Asphodelaceae). *A. greatheadii* grows wild in the northern parts of South Africa, whereas *A. ferox* grows wild primarily in the Eastern and Western Cape provinces. Various extracts of these *Aloe* species are traditionally used and commercially sold as creams, ointments and tonics for the purpose of treating a variety of ailments, of which their applications to arthritis, skin cancer, burns, eczema, psoriasis, digestive problems, high blood pressure and diabetes are most common. There is, however, very little or no scientific evidence to support these claims, with many of the claims being based on research done on *A. vera*. As different *Aloe* species would have varying phytochemical contents due to inter-species variation, and varying climate and soil conditions, direct correlation of biological activity would be inaccurate. Consequently, it is of relevance for scientists, industry and rural communities to not only research the relevant medicinal uses of these indigenous *Aloe* species, but also to determine the active components and their individual or combined mechanisms of biological function.

This paper will primarily focus on identifying, quantifying and comparing the phytochemical composition of two *A. greatheadii* var. *davyana* extracts: a leaf gel extract (LGE) and a 95 % aqueous ethanol leaf gel extract (ELGE), obtained using a modified extraction procedure, and analysis on GC-MS and spectrophotometry. This is done in order to determine whether this plant species contains any individual compound or group of compounds which may substantiate its current commercial and traditionally use as a herbal medicine, in addition to determining the most appropriate methods of extracting these compounds. The results will consequently be discussed in the light of their putative biological or therapeutic relevance.

## Results and Discussion

The individual compounds identified via GC-MS in the LGE and ELGE of *A. greatheadii* var. *davyana* are arranged according to their structural classifications and summarized in Table 1. Of the individual compounds identified, those best described for their health benefits include the polyphenols/phenolic acids, sterols, fatty acids and indoles. Other compounds identified include various alkanes, pyrimidines, alkaloids, organic acids, aldehydes, dicarboxylic acids, ketones and alcohols.

**Table 1:** Concentrations of GC-MS Identified Compounds from Lyophilized A*loe greatheadii* var *davyana* Leaf Gel (LGE) And 95% Aqueous Ethanol Leaf Gel Extracts (ELGE).

Compounds	Concentration (ppm)			Compounds	Concentration (ppm)		
	LGE	ELGE(per dry mass LGE)	ELGE(per dry massELGE)		LGE	ELGE(per dry mass LGE)	ELGE(per dry mass ELGE)
**Organic acids**				**Alcohols**			
Isovaleric	119	71.7	2.60 x 10^3^	1-Propanol	83.9	-	-
Pentanoic	491	40.0	1.5 0x 10^3^	2,3-Butanol	262	-	-
Lactic	2786	111	4.10 x 10^3^	2-Methyl-1,3-propanediol	293	-	-
2-Hydroxyacetic	68.5	-	-	Phenylethanol	51.5	-	-
Pyruvic	23.1	2.56	-	Benzyl alcohol	56.3	133	4.90 x 10^3^
Furancarboxylic	30.3	-	-	2,3-Pentanediol	7.46	-	-
Oxalic	0.88	-	-	Glycerol	1.20	-	-
3-Hydroxypropanoic	0.99	-	-	Octadecanol	11.9	-	-
2-Hydroxyvaleric	83.6	43.7	1.60 x 10^3^	Phytol	20.1	-	-
Cyclohexane-3-carboxylic	0.87	-	-	2-Methyl-1,3-butanol	-	20.6	7.60 x 10^2^
3-Hydroxyisovaleric	110	-	-	Hexanol	23.3	-	-
2-Ketoisovaleric	1.20	39.9	1.50 x 10^3^	Butanol	6.45	-	-
Succinic	415	989	3.70 x 10^4^				
2-Methylsuccinic	61.8	75.1	2.80 x 10^3^	**Aldehydes**			
Methylmalic	10.4	-	-	Benzaldehyde	35.3	156	5.80 x 10^3^
Malic	25.4	126	4.70 x 10^3^	*m*-Tolualdehyde	11.7	-	-
Threonic	1.43	-	-	*p*-Tolualdehyde	-	45.9	1.70 x 10^3^
3,4,5-Trihydroxypentanoic	2.05	-	-	2,3-Dihydroxybenzaldehyde	0.24	-	-
2,3,4,5-Tetrahydroxypentanoic	-	27.9	1.00 x 10^3^	Glyceraldehyde	32.2	-	-
Suberic	6.37	-	-				
3-Hydroxypicolinic	34.9	-	-	**Ketones**			
Isonicotinic	27.6	-	-	2,6-Dimethyl-4-heptanone	153	-	-
2-Ketoglutaric	-	25.5	9.40 x 10^2^	4,6-Dimethyl-2-heptanone	34.5	-	-
Glycolic	-	132	4.90 x 10^3^	Heptanone	-	8.51	3.4 x 10^2^
3-Hydroxypropionic	-	2.31	8.50 x 10^1^				
Methylbenzyl acetate	-	16.7	6.20 x 10^2^	**Pyrimidines**			
Acetic	-	29.2	1.10 x 10^3^	Uracil	554	919	3.40 x 10^4^
Phosphoric	-	233	8.60 x 10^3^	Thymine	428	187	6.90 x 10^3^
Hydantoinpropionic	-	17.5	6.50 x 10^2^				
2-Butoxyethylacetate	-	57.6	2.10 x 10^3^	**Indoles**			
Citric	-	5.94	2.20 x 10^2^	Indole-5-acetic	9.19	-	-
2-Hydroxyglutaric	-	24.8	9.20 x 10^2^	Hexahydrobenzoindole	-	11.4	4.20 x 10^2^
Tartaric	-	9.69	3.60 x 10^2^				
3-Methylvaleric	-	58.6	2.20 x 10^3^	**Alkaloids**			
				Hypoxanthine	33.1	-	-
**Polyphenols / Phenolic compounds**							
Phenol	11.8	46.0	1.70 x 10^3^	**Phytosterols**			
4-Ethylphenol	5.85	-	-	Cholestanol	17.7	-	-
Vanillic	60.7	25.7	9.50 x 10^2^	Campesterol	119	-	-
Homovanillic	23.4	-	-	β-sitosterol	99.6	-	-
Gentisic	55.6	-	-	Stigmasterol	15.8	-	-
6,7-Dihydroxycoumaric	31.3	-	-				
*o*-Hydroxycinnamic	51.3	-	-	**Fatty acids**			
Protocatechuic	162	42.7	1.60 x 10^3^	Lauric (C12:0)	0.35	-	-
3,4-Dihydroxyphenylacetic	2.76	-	-	Tridecanoic (C13:0)	0.02	-	-
Syringic	14.4	-	-	Sebacic (C10:0)	0.01	-	-
Sinapic	37.8	-	-	Myristic (C14:0)	2.86	-	-
Caffeic	107	-	-	Undecanoic (C11:0)	0.03	-	-
Isoferulic	38.4	-	-	Pentadecanoic (15:0)	1.16	-	-
Ferulic	60.1	-	-	Palmitic (C16:0)	43.0	1.49	5.50 x 10^1^
Benzoic	420	3136	1.20 x 10^5^	Stearic (C18:0)	3.24	-	-
Phenylacetic	71.3	283	1.00 x 10^4^	Nonadecanoic (C19:0)	3.14	-	-
2-Methoxybenzoic	233	-	-	Heneicosanoic (C21:0)	0.28	-	-
*o*-Toluic	162	-	-	Behenic (C22:0)	5.39	-	-
Phenylpropionic	37.5	20.3	7.50 x 10^2^	Tricosanoic (C23:0)	1.74	-	-
4-Phenyllactic	613	86.8	3.20 x 10^3^	Lignoceric (C24:0)	5.11	-	-
4-Hydroxybenzoic	223	56.1	2.10 x 10^3^	Arachidonic (C20:4)	0.57	-	-
2,3-Hydroxybenzoic	12.1	-	-	Myristoleic (C14:1)	0.20	-	-
4-Hydroxyphenylacetic	378	45.7	1.70 x 10^3^	10-Pentadecenoic (C15:1)	1.44	-	-
Hydro-*p*-coumaric	13.9	-	-	Palmitoleic (C16:1)	4.00	-	-
*p*-Coumaric	113	-	-	Linoleic (C18:2 n-6)	570	-	-
3-Hydroxyphenylbutyric	-	13.8	5.10 x 10^2^	10-Heptadecenoic (C17:1)	0.48	-	-
4-Hydroxymandelic	-	110	4.10 x 10^3^	Oleic (C18:1)	30.1	-	-
Benzylacetate	-	64.6	2.40 x 10^3^				
2-Hydroxybutyric	-	0.76	2.70 x 10^1^	**Dicarboxylic acids**			
Phenylpyruvic	-	9.41	3.40 x 10^2^	Azelaic	0.04	-	-
				1,2-Benzenedicarboxylic	-	29.5	1.10 x 10^3^
**Alkanes**							
1,3-Dihydroxybutane	8.51	-	-				

“-” denotes nothing detected.

As shown in Table 1, the individual phenolic compounds identified in the highest concentrations in *A. greatheadii* LGE include 4-phenyllactic acid, benzoic acid, 4-hydroxyphenylacetic acid, 4-hydroxy-benzoic acid and 4-methoxymandelic acid. Of the phenolic compounds best known for their health benefits and associated antioxidant properties, 4-hydroxyphenylacetic acid, 2,3-dihydroxybenzoic acid, protocatechuic acid, *p*-coumaric acid and caffeic acid were the most abundant. Of the four phytosterols identified, campesterol and β-sitosterol were by far the most abundant. Only one indole, indole-5-acetic acid, was however identified in the LGE. Comparatively, higher concentrations of the above mentioned polyphenols were also detected in the ELGE (when quantified as per dry mass ELGE). Different to the ethyl acetate/diethyl ether extracts of the LGE, however, hexahydrobenzoindole was the only indole identified in the ELGE, and surprisingly, no phytosterols were detected in this extract.

As shown in Table 2, ORAC and FRAP analyses of LGE and ELGE revealed the ELGE to have greater antioxidant capacity compared to the LGE, which is supported by the higher total polyphenol contents detected in ELGE. The majority of the polyphenols in both the LGE and ELGE, as identified by GC-MS and confirmed spectrophometrically, are non-flavonoids, making-up 83.8 % of the total polyphenol content of the LGE and 92.4 % of the ELGE. An interesting phenomenon was the absence of Aloe-emodin in the *A. greatheadii* extracts. This is of importance as many of the health benefits associated with other *Aloe* species (including similar leaf gel extracts of *A. ferox*, also indigenous to South Africa), are attributed to the presence of, amongst other compounds, Aloe-emodin [7]. Finally, the total sugar contents of these extracts were 5.43 g/100 g for the LGE and 83.76 g/100 g for the ELGE, 36% of which was quantified as glucose, 18% as fructose and the remainder as maltose and sucrose.

We previously reported on the phytochemical composition and antioxidant capacities of *A. ferox* LGE and ELGE [7]. Compared to this species, *A. greatheadii* generally shows less variety and lower concentrations of the above mentioned health-associated antioxidant compounds. Similarly, Table 2 indicates the total polyphenol and non-flavonoid contents, as well as the antioxidant capacities of *A. greatheadii* LGE and ELGE, as measured by FRAP, to be lower than that as previously reported for *A. ferox*. The total flavonoid contents and the antioxidant capacities of *A. greatheadii* LGE, as measured by ORAC, were however higher comparatively, which may be indicative of the types of polyphenols in *A. greatheadii* having stronger scavenging ability than ferric ion reducing potential. Apart from inter-species variation explaining these differences, soil conditions and climate may also play a role in the varying phytochemical contents of these plants.

**Table 2:** Concentrations of Total Polyphenols, Flavonoids, Non-Flavonoids, Oxygen Radical Absorbance Capacity (ORAC) and Ferric Reducing Antioxidant Power (FRAP) in Lyophilized *Aloe greatheadii* var *davyana* Leaf Gel (LGE) and 95% Aqueous Ethanol Leaf Gel Extracts (ELGE).

Compound	LGE(dry mass)	LGE(wet mass)	ELGE(expressed as dry mass ELGE)	ELGE(expressed as dry mass LGE)	ELGE(expressed as wet mass LGE)
Total polyphenols (mg of GAE/100g ± SD)	45.1 ± 0.94	1.20 ± 0.03	263 ± 6.51	30.9 ± 0.77	0.82 ± 0.02
Total flavonoids (mg of CE/100g ± SD)	7.66 ± 0.26	0.20 ± 0.01	20.2 ± 0.50	2.37 ± 0.06	0.06 ± 0.001
Total non-flavonoids (by calculation)	37.8 ± 0.99	0.99 ± 0.03	243 ± 6.96	28.6 ± 0.82	0.75 ± 0.02
ORAC – hydrophyllic (μmol of TE/g)	59.0 ± 1.16	2.05 ± 0.4	83.0 ± 1.32	5.42 ± 1.21	0.19 ± 0.04
ORAC – lipophyllic (μmol of TE/g)	-	-	-	-	-
ORAC – total (μmol of TE/g)	59.0 ± 1.16	2.05 ± 0.4	83.0 ± 1.32	5.42 ± 1.21	0.19 ± 0.04
FRAP (μmol/g)	2.63 ± 0.21	0.09 ± 0.01	8.98 ± 0.21	0.58 ± 0.01	0.02 ± 0.001

“-” denotes nothing detected.

From an analytical perspective, GC-MS analyses identified a larger number of compounds using direct LGE ethyl acetate/diethyl ether and hexane extractions, as compared to using the same extraction procedures on the ELGE, when quantified as per dry mass LGE (Table 1). This indicates that direct LGE ethyl acetate/diethyl ether and hexane extractions are best suited for general phytochemical characterization purposes. However, despite there being fewer compounds identified in the ELGE, the concentrations for many of the compounds with associated health benefits, and total sugars, were found to be between 1.2 to 1,250 times higher than the same compounds identified in the LGE, when expressed as per dry mass ELGE. This was further confirmed by the antioxidant capacity analyses showing higher activities in the latter extracts. This justifies the use of the latter approach for preparing extracts for use in *in vivo* or *in vitro* biological efficacy and mechanistic studies.

Epidemiological evidence supports the hypothesis that the consumption of foods rich in natural antioxidants plays an important role in the prevention of several chronic diseases associated with oxidative stress, including diabetes, cancer, hypertension and cardiovascular diseases [8]. Due to the fact that the majority of the phytochemicals identified in *A. greatheadii* were polyphenols/phenolic acids, one would expect these to be the major contributors to this plant’s antioxidant capacity and its proposed use for alleviating or preventing diseases associated with oxidative stress [9,10,11].

Considering that the mechanism proposed for developing diabetes is hyperglycaemia induced oxidative stress [5,12,13,14], the current use of *A. greatheadii* in traditional and commercial tonics for treating this disease may be justified. To date, various authors have reported on the anti-diabetic properties of a variety of other *Aloe* species from both human and animal trials, of which *Aloe vera* and *Aloe arborenscens* are by far the best described. Interventions involving various extracts, including a 95 % aqueous ethanol extract of these, have been shown to alleviate the diabetic state. By preventing hyperglycaemia induced oxidative stress and the associated pancreatic β-cell destruction, these plant extracts have been shown to increase insulin secretion by the pancreas, and in so doing correct the diabetes associated hyperglycaemia and dyslipidaemia [5,12,13,14]. This action is ascribed to the various polyphenols present in these extracts, which alleviate the diabetic condition by lowering glucose uptake, and in doing so prevent hyperglycaemia [15,16]. Additionally, the plant sterols identified in *A. greatheadii* also possess similar glucose lowering effects. Tanaka *et al*. (2006) reported reductions in both fasting and random blood glucose levels of *db/db* diabetic mice chronically treated with the same phytosterols from *A. vera* leaf gel [17]. Apart from these glucose lowering effects, phytosterols are better known for their total cholesterol and low-density lipid cholesterol (LDL-C) lowering effects [18]. As summarized by Devaraj and Jialal [19], evidence for this has been observed in hypercholesterolemic, diabetic and healthy volunteers [19]. The mechanism proposed by which phytosterols accomplish this, is by lowering cholesterol absorption due to the structural similarities that these compounds share with cholesterol [20,21,22]. Apart from lowering cardiovascular risk factors associated with diabetes and other diseases, β-sitosterol has been shown to positively influence a diabetic state by directly lowering fasting blood glucose levels by cortisol inhibition [23]. Furthermore, phytosterols have been shown to reduce biomarkers for oxidative stress and inflammation [19], as well as to reduce cancer development by a variety of mechanisms [24]. Based on the presence of the high amounts of many of the same polyphenols and phytosterols in *A. greatheadii* LGE and ELGE, this *Aloe* species may show promise in preventing or alleviating the progression of diseases associated with oxidative stress, including diabetes, cancer, hypertension and cardiovascular diseases. On the other hand, the total sugar/glucose contents of these extracts may be of concern, especially when considering using these extracts in the context of a diabetes intervention, as carbohydrate intake is considered a major factor in glycaemic control. Nielsen and co-workers report that a low carbohydrate diet, containing 20 % carbohydrates, is superior to a diet containing 55 - 60 % carbohydrates, with regards to controlling bodyweight, blood glucose levels and reducing in HbA_1c_ [25]. The American Diabetes Association (ADA) defines a low carbohydrate diet as less than 130 g/d or 26 % of a nominal 2,000 kcal (8,400 kJ) diet [26]. Considering the above mentioned recommended carbohydrate intakes for diabetics and the mechanisms by which the pholyphenols and phytosterols elicit their anti-diabetic actions (by lowering glucose absorption and protecting pancreatic β-cells from oxidative destruction), the sugar contents of these extracts may not necessarily be problematic due to the small amounts that would be additionally ingested during an intervention using these extracts. This however should be investigated, in addition to other methods of extraction which could potentially eliminate these sugars.

## Conclusions

Analytically, direct LGE ethyl acetate/diethyl ether and hexane extractions produce better phytochemical characterization, whereas 95 % aqueous ethanol extraction concentrates a number of health related compounds, justifying its applications to *in vivo* efficacy studies. From a medicinal application perspective, *A. greatheadii* contains a variety of compounds (esp. polyphenols and phytosterols) with confirmed antioxidant capacity, and putative therapeutic actions (including blood glucose, cholesterol and cortisol lowering properties) relating to the prevention or treatment of diabetes, cardiovascular disease, cancer and hypertension. No toxic compounds were detected in these *Aloe* extracts, however, due the presence of other confounders which may have been missed in this study, further confirmation of the proposed health benefits of these extracts through *in vivo* animal experimentation is strongly suggested.

## Experimental

### General

All analytical standards and reagents used for quantification, and those used for generating mass spectra for GC-MS identification, were purchased from Sigma-Aldrich (St Louis, MO, USA). All organic extraction solvents used were of ultra high purity purchased from Burdick and Jackson (USA). Folin-Ciocalteu’s phenol reagent and other reagent chemicals were purchased from Merck (Darmstadt, Germany).

### Plant material

Whole, freshly cut, *A. greatheadii* var. *davyana* leaves (100 kg) were harvested from approximately 200 plants in the month of May (2007) from a rural area in the Potchefstroom district of the North West Province in South Africa (herbarium deposit site: AP Goossens Herbarium (code: PUC), Potchefstroom South Africa; voucher number : PUC 7951). All leaves were collected from mature plants with a circular diameter greater than 50 cm.

### Sample preparation

The leaf skin was removed by hand and the leaf gel homogenized, lyophilized and stored at -20 ^o^C until analysis. This was termed the leaf gel extract (LGE). A large portion of the LGE was used for the preparation of a 95 % aqueous ethanol leaf gel extract (ELGE). Batches (420 g) of finely ground lyophilized *Aloe* gel were extracted using 95 % aqueous ethanol (500 mL), followed by sonication for 10 min and shaking for 1 hour. The solvent was collected following centrifugation at 3000 x *g* for 10 min. This was repeated 10 times to ensure total extraction of all compounds from the lyophilized extracts. The supernatants were pooled and evaporated to dryness under reduced pressure in a rotary evaporator. The residue was stored in dry sterilized containers at -20 ^o^C until further use.

### Ethyl acetate/diethylether extraction

3-Phenylbutyric acid (5 mg/mL, 100 µL) was added as an internal standard to LGE and ELGE (25 mg) followed by sodium acetate buffer (0.125 M, 1 mL) and β-glucuronidase (60 µL). Samples were then incubated at 37 ^o^C for 3 hours. Incubated samples were subsequently extracted with ethyl acetate (6 mL) followed by diethyl ether (3 mL). After centrifugation, collected supernatants were pooled and dried under a nitrogen stream. Derivatization with bis(trimethylsilyl) trifloroacetamide (BSTFA, 100 µL), trimethylchlorosilane (TMCS, 20 µL) and pyridine (20 µL) at 70 ^o^C for 30 min followed. After cooling to room temperature, 0.1 µL of the derivatized extract was injected into the GC-MS via splitless injection.

### Hexane Extraction for Fatty Acids

The internal standard, heptadecanoic acid (72 mM), was added to LGE and ELGE (25 mg) followed by a 45 mM solution of butylated hydroxytoluene (100 µL) and methanolic HCL (3N, 1 mL). The samples were vortexed and incubated for 4 hours at 90 ºC. After cooling to room temperature, the samples were extracted twice with 2 ml of hexane, dried under a nitrogen stream and finally re-suspended with hexane (100 µL), 0.1 µL of which was injected onto the GC-MS via splitless injection.

### Phytochemical Characterization via GC-MS

An Agilent 6890 GC ported to a 5973 MS detector (California, USA) was used for identification and quantification of individual phytochemicals, using MS libraries previously compiled from purchased standards. For the acquisition of an electron ionization mass spectrum, an ion source temperature of 200 °C and electron energy of 70 eV was used. The GC was equipped with a SE-30 capillary column (Chemetrix, USA), a split/splitless injection piece (250 °C) and direct GC-MS coupling (260 °C). Helium (1 mL/min) was used as the carrier gas. The oven temperature program for analyzing the ethyl acetate/diethylether extracts utilized an initial oven temperature of 40 ^o^C, maintained for 2 min, followed by a steady climb to 350 ^o^C at a rate of 5 ^o^C/min. For the fatty acid analysis, hexane extracts were analyzed using an initial oven temperature of 50 ^o^C, maintained for 1.5 min, and then allowed to increase to 190 ^o^C at a rate of 30 ^o^C/min. This oven temperature was again maintained at 190 ^o^C for 5 min and then allowed to increase to 220 ^o^C at a rate of 8 ^o^C/min. This oven temperature was maintained for 2 min and finally ramped to 230 ^o^C at a rate of 3 ^o^C/min and maintained for a further 24 min.

### Total Polyphenols

The total polyphenol contents of the LGE and ELGE were determined according to the Folin-Ciocalteu procedure [27]. Briefly, finely ground LGE or ELGE (10 mg) was dissolved in H_2_O (200 μL) in a test tube and Folin-Ciocalteu’s reagent (1 mL) was added. This was allowed to stand for 8 min at room temperature. Next, sodium carbonate (7.5 %, w/v, 0.8 mL) was added, mixed and allowed to stand for 30 min. Absorption was measured at 765 nm (Shimadzu UV-1601 Spectrophotometer). The mean total phenolic contents (n = 3) were expressed as milligram gallic acid equivalents per 100 g (mg GAE/100g dry mass or wet mass ± standard derivatives).

### Total flavonoids

The total flavonoid contents of the LGE and ELGE were measured using the aluminium chloride assay as described by Zhishen and co-workers with slight modifications [28]. Briefly, LGE or ELGE (10 mg) was dissolved in H_2_O (1 mL) in a test tube, to which 5 % (w/v) NaNO_2_ (60 µL) was added. After 5 min, a 10% (w/v) AlCl_3_ solution (60 µL) was added. After 6 min, 1 M NaOH (400 µL) was added and the total volume made up to 2 mL with H_2_O. The solution was mixed well and the absorbance measured at 510 nm against a reagent blank. Concentrations were determined using a catechin standard curve. Mean total flavonoid contents (n = 3) were expressed as milligrams catechin equivalents (CE) per 100 g (mg CE/100g dry or wet mass ± standard deviation).

### Oxygen Radical Absorbance Capacity (ORAC)

ORAC analyses of hydrophilic and lipophylic fractions of the LGE and ELGE were performed essentially as described by Prior *et al*. [29]. The analyses of lipophylic compounds were aided by the addition of randomly methylated β-cyclodextrin (kind gift from Dr R Prior) as a solubility enhancer as described by Huang *et al*. [30]. Briefly, in a volume of 200 μL, the reaction contained 56 nM fluorescein as a target for free radical attack by 240 nM 2,2’-azobis(2-amidinopropane) dihydrochloride. A fluorescence plate reader (BioTEK FL-600, UK) was used and the decay of fluorescence of fluorescein (excitation 485 nm, emission 520 nm) was measured every 5 min for 2 hours at 37 °C. Costar black opaque (96-well) plates were used in the assays. Trolox was used as a standard at a range of between 0-20 µM, giving a polynomial (2nd order) curve fit analysis. Mean values (n = 3) of antioxidant capacities were expressed as µmole trolox equivalents (TE) / g wet and dry mass ± standard deviation.

### Ferric Reducing Antioxidant Power (FRAP)

FRAP values of the LGE and ELGE were determined essentially as described previously [31]. Briefly, the reduction of a Fe^3+^-2,3,5-triphenyltetrazolium complex in the assay, by the antioxidants in the samples, was monitored at 593nm. As a standard, FeSO_4_ was used and the FRAP activities of the samples were expressed as the mean (n = 3) μmol Fe^2+^/g wet and dry mass ± standard deviation.

### Sugar content

The total sugar content as well as the type of sugar present in the LGE and ELGE was determined at the Department of Agriculture, Directorate Food Safety and Quality Assurance, Division Analytical Services North, Pretoria, South Africa. The total sugar content was determined by Refractive index (RI) as previously described [32]. The concentrations of fructose and glucose were determined via HPLC using a Supelcosil LC-NH2 column (Supelco, 250 x 4.6 mm, 5 µm, Sigma-Aldrich Catalogue No: 58338) and 75% Acetonitrile as mobile phase with a flow rate of 1.5 mL/min at 30^o^C and detected by a Refractive Index (RI) detector. The results are expressed as percentage sugars (m/m) in LGE and ELGE.

## References

[1] Cunningham A.B. (1993). African medical plants: setting priorities at the interface between conservation and primary healthcare. People and plants working paper 1.

[2] Thring T.S.A., Weitz F.M. (2006). Medicinal plant use in the Bredasdorp/Elim region of the Southern Overberg in the Western Cape Province of South Africa. J. Ethnopharmacol..

[3] Morton J.F. (1961). Folk uses and commercial exploitation of aloe leaf pulp. Econ. Botany.

[4] Crosswhite F.S., Crosswhite C.D. (1984). Aloe vera, plant symbolism and the threshing floor. Desert Plants.

[5] Reynolds T., Dweck A.C. (1999). Aloe vera leaf gel: a review update. J. Ethnopharmacol..

[6] Rajasekaran S., Sivagnaman K., Subramanian S. (2005). Modulatory effects of Aloe vera leaf gel extract on oxidative stress in rats treated with streptozotocin. J. Pharm. Pharmacol..

[7] Loots Du T., Van der Westhuizen F.H., Botes L. (2007). *Aloe ferox* leaf gel phytochemical content, antioxidant capacity and possible health benefits. J. Agric. Food. Chem..

[8] Willcox J.K., Ash S.L., Catignami G.L. (2004). Antioxidants and the prevention of chronic disease. Crit. Rev. Food. Sci. Nutr..

[9] Cao G., Sofic E., Prior R.L. (1997). Antioxidant and prooxidant behavior of flavonoids: structure-activity relationships. Free. Radic. Biol. Med..

[10] Herraiz T., Galisteo J. (2004). Endogenous and Dietary Indoles: A Class of Antioxidants and Radical Scavengers in the ABTS Assay. Free. Radic. Res..

[11] Scalbert A., Manach C., Morand C., Re´Me´ Sy C. (2005). Dietary polyphenols and the prevention of disease. Crit. Rev. Food. Sci. Nutr..

[12] Agarwal PO. (1985). Prevention of atheromatous heart disease. Angiology.

[13] Beppu H.T., Koike K., Shimpo T., Chihara M., Hoshino C.I., Kuzuya H. (2003). Radical-scavenging effects of Aoe arborescens Miller on prevention of pancreatic islet β-cell destruction in rats. J. Ethnoparmacol..

[14] Rajasekaran S., Sivagnaman K., Subramanian S. (2005). Antioxidant effect of Aloe vera gel extract in streptozotocin-induced diabetes in rats. Pharmacol. Rep..

[15] Cao H., Hininger-Favier I., Kelly M.A., Benaraba R., Dawson H.D., Coves S., Roussel A.M., Anderson R.A. (2007). Green tea polyphenol extract regulates the expression of genes involved in glucose uptake and insulin signalling in rats fed a high fructose diet. J. Agric. Food. Chem..

[16] Johnston K., Sharp P., Clifford M., Morgan L. (2005). Dietary polyphenols decrease glucose uptake by human intestinal Caco-2 cells. Fed. Eur. Biochem. Soc. Lett..

[17] Tanaka M., Misawa E., Ito Y., Habara N., Nomaguchi K., Yamada M., Toida T., Hayasawa H., Takase M., Inagaki M., Higuchi R. (2006). Identification of five phytosterols from aloe vera gel as anti-diabetic compounds. Biol. Pharm. Bull..

[18] Patch C.S., Tapsell L.C., Williams P.G., Gordon M. (2006). Plant sterols as dietary adjuvants in the reduction of cardiovascular risk: theory and evidence. Vasc. Health. Risk. Manag..

[19] Devaraj S., Jialal I. (2006). The role of dietary supplementation with plant sterols and stanols in the prevention of cardiovascular disease. Nutr. Rev..

[20] Lichtenstein A.H., Deckelbaum R., AHA Science Advisory (2001). Stanol/sterol ester-containing foods and blood cholesterol levels. A statement for healthcare professionals from the Nutrition Committee of the Council on Nutrition, Physical Activity, and Metabolism of the American Heart Association. Circulation.

[21] Normen L., Dutta P., Lia A., Andersson H. (2000). Soy sterol esters and beta-sitostanol ester as inhibitors of cholesterol absorption in human small bowel. Am. J. Clin. Nutr..

[22] Jones P.J., Ntanios F.Y., Raeini-Sarjaz M., Vanstone C.A. (1999). Cholesterol-lowering efficacy of a sitostanol-containing phytosterol mixture with a prudent diet in hyperlipidemic men. Am. Clin. Nutr..

[23] McAnuff M.A., Harding W.W., Omoruyi F.O., Jacobs H., Morrison E.Y., Asemota H.N. (2005). Hypoglycemic effects of steroidal sapogenins isolated from Jamaican bitter yam, Dioscorea polygonoides. Food. Chem. Toxicol..

[24] Bradford P.G., Awad A.B. (2007). Phytosterols as anticancer compounds. Mol. Nutr. Food. Res..

[25] Nielsen J.V., Jönsson E.A. (2006). Low-carbohydrate diet in type 2 diabetes. Stable improvement of body weight and glycaemic control during 22 months follow-up. Nutr. Metab. (Lond)..

[26] American Diabetes Association (2007). Nutrition recommendations and interventions for diabetes. Diabet. Care.

[27] Singleton V.L., Rossi J.A. (1965). Colorimetry of total phenolics with phosphomolybdic-phosphostungastic acid reagents. Am. J. Enol. Vitic..

[28] Marinova D., Robarova F., Atanassova M. (2005). Total phenolics and total flavonoids in bulgarian fruits and vegetables. J. Univ. Chem. Technol. Metall..

[29] Prior R.L., Hoang H., Gu L., Wu X., Bacchiocca M., Howard L., Hampsch-Woodill M., Huang D.M., Ou B., Jacob R. (2003). Assays for hydrophilic and lipophilic antioxidant capacity (ORAC(FL)) of plasma and other biological and food samples. J. Agric. Food. Chem..

[30] Huang D., Ou B., Hampsc-Woodill M., Flanagan J.A., Deemer E.K. (2002). Development and validation of oxygen radical absorbance capacity assays for lipophilic antioxidants using randomly methylated beta-cyclodextrin as the solubility enhancer. J. Agric. Food. Chem..

[31] Benzie I.F., Strain J.J. (1999). Ferric reducing/antioxidant power assay: direct measure of total antioxidant activity of biological fluids and modified version for simultaneous measurement of total antioxidant power and ascorbic acid concentration. Meth. Enzymol..

[32] Stevens J.W., Baier W.E. (1939). Corrections for obtaining soluble solids from refractometer readings (soluble solids as sucrose). Ind. & Eng. Chem..

